# Glucose Regulated Protein 78 Phosphorylation in Sperm Undergoes Dynamic Changes during Maturation

**DOI:** 10.1371/journal.pone.0141858

**Published:** 2015-11-30

**Authors:** Vivian Lobo, Parimala Rao, Rahul Gajbhiye, Vijay Kulkarni, Priyanka Parte

**Affiliations:** 1 Department of Gamete Immunobiology, National Institute for Research in Reproductive Health (ICMR), Mumbai, 400012, India; 2 Department of Reproductive Endocrinology and Infertility, National Institute for Research in Reproductive Health (ICMR), Mumbai, 400012, India; Nagoya University, JAPAN

## Abstract

GRP78, a resident endoplasmic reticulum (ER) chaperone involved in protein transport, folding and assembly, has been reported in sperm. It is shown to be localized in the neck region of human sperm. We have previously reported GRP78 to be less phosphorylated in asthenozoosperm.The present study aimed to determine whether sperm GRP78 undergoes phosphorylation changes during epididymal maturation and whether there are any differences in GRP78 phosphoforms in asthenozoosperm vis-à-vis normozoosperm. Testicular- and cauda epididymal- sperm from adult male Holtzman rats, and semen ejaculates collected from normal and asthenozoospermic individuals were investigated. DIGE carried out to determine phosphorylation of GRP78 in asthenozoosperm and normal sperm reveals a shift in the location of GRP78 of asthenozoosperm towards the alkaline pH, indicative of reduced GRP78 phosphorylation. Immunoprecipitation studies using antibodies specific to GRP78, serine-, threonine-, and tyrosine phosphorylation and Pan phospho antibody demonstrates GRP78 to be phosphorylated at all three residues in rat spermatozoa. Phosphatase assays using Calf intestinal alkaline phosphatase and Lambda protein phosphatase followed by nanofluidic proteomic immunoassay (NIA) show that in rat, GP_4.96_, GP_4.94_ and GP_4.85_ are the three phosphoforms in mature (caudal) sperm as against two phosphoforms GP_4.96_and GP_4.94_in immature (testicular) sperm. In mature human sperm GP_5.04_, GP_4.96_, and GP_4.94_were the 3 phosphoforms observed. GP_4.94_[P = 0.014]andGP_5.04_ [P = 0.02] are significantly reduced in asthenozoosperm. Ours is the first report indicating GRP78 in sperm to be phosphorylated at serine, threonine and tyrosine residues contrary to published literature reporting GRP78 not to be tyrosine phosphorylated. We report the presence of GRP78 phosphoforms in rat- and human- sperm and our data suggest that GRP78 phosphorylation in sperm undergoes spatial reorganization during epididymal maturation. Significant differences observed in 2 out of 3 phosphoforms in asthenozoosperm suggest that GRP78 phosphorylation may have functional relevance in sperm with consequent clinical implications.

## Introduction

Glucose Regulated Protein 78 (GRP78), a member of heat shock protein 70 family is a calcium sensitive chaperone induced upon calcium or glucose stress or energy deprivation and also an anti-apoptotic protein [1, 2]. Other than its chaperonic functions, GRP78 located on the cell surface is implicated in mitogenesis and cellular proliferation [3] and serves as a receptor for the uptake of certain viruses [4]. GRP78 also functions as membrane receptor for activated α-2-macroglobulin (α2M*) [3, 5]. Surface expression of GRP78 has also been reported on human sperm [6].

In the male reproductive system, GRP78 is demonstrated in testis, epididymis and sperm [7, 8]. Gene and protein expression of GRP78 has been reported in pachytene and round spermatids during spermatogenesis [9]. Exogenously added recombinant GRP78 reportedly binds to the sperm surface and increase intracellular calcium concentration; but does not affect sperm viability, motility and acrosomal integrity [10]. Whilst Lachances’ (2010) group observed no correlation betweenGRP78 and severity of asthenozoospermia, Shen and co-workers identified it to be differentially expressed in idiopathic asthenozoospermic individuals compared to normal individuals [11]. Our studies investigating the phosphoproteins relevant to sperm motility demonstrated that whilst total GRP78 protein did not differ between normozoosperm and asthenozoosperm, it was significantly less phosphorylated in asthenozoosperm [12]. The present study was initiated to answer two questions; 1) Does sperm GRP78 undergo phosphorylation changes during epididymal maturation, 2) Are there any differences in GRP78 phosphoforms in asthenozoosperm vis-à-vis normozoosperm.

Using the Differential In Gel Electrophoresis (DIGE) approach, we demonstrate a shift in the electrophoretic mobility of phosphorylated GRP78 in asthenozoosperm vis-à-vis normozoosperm. Our data also shows that GRP78 is phosphorylated at serine-, threonine-, and tyrosine- residues. We further demonstrate that in the rat, three phosphorylated forms of GRP78 are present in the caudal (mature) sperm as against two phosphorylated forms in testicular (immature) sperm. In normal human sperm, phosphorylated GRP78 exists in 3 forms, two of which are significantly reduced in asthenozoosperm. Our findings suggest that 1) GRP78 phosphorylation in sperm undergoes a transformation during sperm maturation; 2) Two out of three GRP78 phosphoforms in human are significantly reduced in asthenozoosperm suggesting that these forms may have functional relevance in sperm motility.

## Materials and Methods

### Study Approval

The study using human semen samples was approved by National Institute for Research in Reproductive Health Ethics Committee for Clinical Studies (ICEC), Mumbai, India. Before sample collection, written informed consent was obtained from the participants.All animal care practices and experimental procedures complied with the guidelines of the Care and Prevention Society against Cruelty of Experimental Animals (CPCSEA) and were approved by the Institutional Animal Ethics Committee (IAEC) of National Institute for Research in Reproductive Health.

### Animal model used

Three month old adult Holtzman male rats (90–110 days) and weighing ~ 225–260 gmwere used. Animals were maintained at a temperature of 22–23°C, humidity of 50–55% and a cycle of 14h light 10h dark with food and water available *ad libitum*.

### Human semen samples

Semen ejaculates were collected from 6 normal and 6 asthenozoospermic individuals by masturbation into sterile plastic containers and allowed to liquefy for 30min at 37°C. For every individual, two ejaculates were collected at an interval of three weeks. Semen analysis was performed according to World Health Organization guidelines[13], the inclusion-exclusion criteria for the recruitment of individuals with normal sperm or asthenozoosperm were as reported previously by us [12].Semen samples obtained from the individuals were subjected to Percoll separation[14] and the sperm so obtained were washed and processed as described further.

### Differential in Gel Electrophoresis (DIGE)

**Fig 1:**
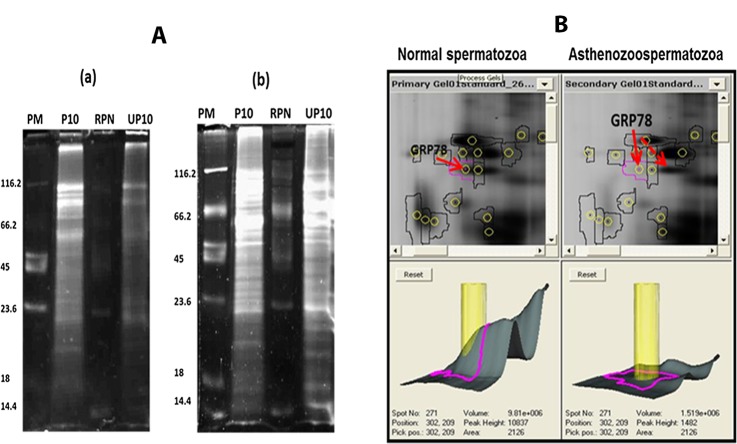
Differential expression of phosphorylated GRP78 in asthenozoosperm. A) Phosphoprotein enrichment. 10μg of phosphorylated (P10) and unphosphorylated (UP10) fractions of sperm lysates were electrophoresed on 1D SDS-PAGE and stained with Pro-Q Diamond Phosphoprotein gel stain which stains only phosphoproteins (**a**) and subsequently with SYPRO® Ruby which stains all proteins (**b**). This phosphoprotein enrichment was done for both normal and asthenozoospermic samples. PM: Peppermint phospho marker, RPN756: was the protein marker used (GE Healthcare Life Sciences, UK). **B) A representative DIGE gel showing the profile of a few proteins in normal sperm and asthenozoosperm.** The image on the left represents a snapshot of a fewphosphoproteins from normal sperm (Cy 3 labelled) and one on the right represents the same snapshot but that of asthenozoosperm (Cy 5 labelled). The arrow indicates identity of the spot as GRP78. Dotted arrow in the snapshot for asthenozoosperm points to the shifted location of phosphorylated GRP78 to the basic side suggesting less phosphorylation as compared to normal.

DIGE was performed following the protocol described by Yan’s group [15]. Sperm from normal- and asthenozoospermic individuals were processed for Differential In Gel Electrophoresis (DIGE) after phosphoprotein enrichment performed exactly as described earlier [12]. The protein content was quantified using the modified BCA assay. Enrichment of the phosphorylated proteins was confirmed by electrophoresing10μg of phosphorylated and unphosphorylated fractions on 1D SDS-PAGE gels and staining the gel with Pro-Q Diamond Phosphoprotein gel stain (Invitrogen, CA, USA) and subsequently with SYPRO® Ruby (Molecular probes, Eugene, OR) (Fig 1A). 50μg phosphoprotein from each normal and asthenozoospermic individual were labeled with 100pmol of Cy3 and Cy5, respectively. Internal standard was prepared by pooling equal amount of protein from each of the samples in the experiment and 50μg of this was labeled with Cy2 dye, as per the kit protocol (Cy dye minimal labeling kit, GE Healthcare). The Cy 3 -, Cy 5 -, and Cy 2—labeled samples were then pooled and passively rehydrated on a 13 cm 3–10 NL IPG strip and focused using a EttanIPGPhor II focusing unit (GE Healthcare) to resolve the proteins on the basis of their isoelectric point (pI). Towards this, active rehydration was done at 50V for 10h, pre-focusing at 200V, 500V, 1000V, and 2500V for 1h each, followed by isoelectric focusing at a gradient 6000V for 20000Vh, and 7000V for 0.5h. Following focusing, the IPG strip was equilibrated in 0.5% w/v DTT and 2.5% w/v iodoacetamide, sequentially in equilibration buffer containing 6M urea, 2% SDS, 50 mM Tris, pH 8.4, and 30% glycerol. The proteins on the strips were further resolved on the basis of their molecular size on 12% SDS-PAGE. The images were acquired on Ettan DIGE imager at excitation wavelengths of 532nm for Cy3, 633nm for Cy5 and 488nm for Cy2. All gels were scanned at a resolution of 100μm. Analysis of the differentially expressed protein spots was done using the DeCyder 6.5 software (GE Healthcare). As the phosphorylated fractions were too little to run preparatory gels, protein spots of interest were picked robotically (Investigator^TM^ProPic II, Genomic Solutions, UK) from the analytical gels, processed for trypsin digestion, and analyzed by MALDI-TOF-MS/MS in the reflectron mode using a 4700 Proteomics Analyzer mass spectrometer (Applied Biosystems, Illinois, US). The protocol followed was as described earlier [16]. Briefly, protein spots of interest (as identified from DIGE analysis using DeCyder Software) were picked robotically from the analytical gels and digested in-gel with trypsin protease and extracted with trifluoroacetic acid (TFA) and acetonitrile (ACN). The spots were alternately treated with 25mM ammonium bicarbonate and 50% ACN, twice followed by a final treatment with 100% ACN and incubated with 150ng of trypsin overnight at 37°C. The in-gel digested peptides were eluted with grades of 0.1%, 0.5%, and 1% TFA in 50% ACN. Approximately 0.5μl tryptic peptide was mixed with 0.5μl α-cyano-4-hydroxycinnamic acid matrix, and 0.5μl of this mixture was spotted onto the target plate. MALDI-TOF-MS/MS was performed in the reflectron mode. Combined MS and MS/MS spectra were subjected to protein identification against the taxonomy Mammalia (mammals) in the Swiss-Prot Protein knowledgebase (Swiss-Prot Release 20080226; 356194 sequences; 127836513 residues) and / NCBInrDataBase (Release 20070216; 4626804 sequences; 1596079197 residues) using the GPS software (version 3.5, Applied Biosystems) running Mascot search algorithm (version 2.0, Matrix Science, Boston, MA) for peptide and protein identification. For peptide matching, a maximum of one miscleavage per peptide and peptide modifications by oxidation of Met and carbamidomethylation of Cys were allowed. The peptide mass tolerance and ion mass (MS/MS) accuracy used for peptide matching were 100 ppm and 0.25 Da, respectively. The confidence of peptide matches was based on the significant value of the MOWSE score and the percentage of sequence coverage.

### Rat sperm isolation and processing

Adult male Holtzman rats were sacrificed by CO_2_ asphyxiation. Testes and caudal region of the epididymis were dissected separately and incised in 0.1M PBS and incubated at 34°C for 30min on a shaker. The sperm released on incubation were pelleted by centrifugation at 800g for 30min at 4°C and the pellets washed thrice with 0.1M PBS. Testicular sperm pellets were further processed by Percoll gradient separation to enrich testicular sperm [17]. Sperm (testicular and caudal) were then suspended either in NP40 lysis buffer containing protease inhibitor cocktail (Roche Diagnostics, Indianapolis, USA), phosphatase inhibitor cocktail (Sigma Aldrich, St. Louis, MO) and 1% Triton X-100 (Sigma Aldrich, St. Louis, MO), overnight for protein extraction or in 4% PFA-PBS for Indirect immunofluorescence.

Sperm pellets suspended in NP40 lysis buffer [50mM Tris HCl (pH 7.4), 150mM NaCl, 0.5% NP40 detergent, 1% Triton X 100, 1mM EDTA, 1mM EGTA (pH 8), 0.2mM PMSF, 0.2mM sodium orthovanadate] as above were homogenized on iceat 14,000rpm, 5min (30s homogenization, 30s cooling; 10 cycles) using a polytron homogenizer (KINEMATICA, Lucerne, Switzerland). The lysates thus obtained were centrifuged at 16,000g, 4°C, for 45min, the protein content of the supernatant quantified by Bradford’s method [18] and used for Western blot, Immunoprecipitation and NIA.

### Indirect immunofluorescence (IIF)

Mature (caudal) rat sperm and ejaculated normal human sperm fixed in 4% paraformaldehyde were used for Indirect Immunofluorescence studies. Post fixation, the sperm were pelleted by centrifugation at 800gfor 10min at 4°C, washed thrice in 0.1M PBS and resuspended in 0.1M PBS. 10μl of this sample was smeared on poly-L-Lysine coated slides. Sperm were either permeabilized with 1% Triton X- 100 (Sigma Aldrich, St. Louis, MO, USA) and 0.5% glacial acetic acid in 0.1M PBS for 10min at RT or left unpermeabilized. They were then washed thrice with 0.1M PBS for 5min and non-specific binding was blocked by incubating the smears in0.5% BSA at RT for 1h in a humid chamber. The sperm smears were then incubated overnight at 4°C with 1:100 diluted rat anti- GRP78 antibody raised in rabbit (Sigma Aldrich, St. Louis, MO, USA). The following day, slides were washed thrice with 0.1M PBS and then incubated with FITC conjugated Goat anti Rabbit IgG (1:100) at RT for 1h in the dark. Slides were washed as mentioned above and mounted in ProLong Gold antifade agent (Invitrogen, OR, USA) and examined ona confocal fluorescent microscope (Zeiss, Jena, Germany). Smears incubated with buffer only in place of the primary antibody served as ‘Negative control’. 4′, 6-diamidino-2-phenylindole (DAPI) (300nM) (Roche, Mannheim, Germany) was used to stain the nuclei.

### Western blot analysis

40μg of sperm protein lysates were electrophoresed in a 12% SDS-PAGE, transblotted on a Nitrocellulose (NC) membrane (Hybond-C Extra) at 100V for 1.25h. The blots were stained with Ponceau S to ensure protein transfer, washed and blocked with 5% NFDM for 1h at RT with gentle rocking. Post blocking, membranes were incubated overnight with anti GRP78 antibody (1:1000),followed by incubation with 1:3000 diluted HRP conjugated swine anti-rabbit IgG (DakoCytomation, Glostrup, Denmark) for 1h at RT and then washed thrice with 0.1% PBS-T for 5 min. The signals on the blot were detected by chemiluminescence using ECL Plus kit (GE Healthcare, Buckinghamshire, UK). Protein molecular weight markers were procured from Fermentas (MD, USA). Blots incubated with antibody dilution buffer in place of the primary antibody served as ‘Negative control’.

### Immunoprecipitation

IP experiments were performed to pull down GRP78 which was probed with anti GRP78 and Pan phospho antibody. In a vice versa experiment, serine-, threonine- and tyrosine- phosphorylated proteins were pulled down using agarose beads conjugated with their respective antibodies and these were probed with anti GRP78 antibody.

#### Immunoprecipitated GRP78 probed with Pan Phospho antibody

Briefly,100μg of rat testicular- and caudal- sperm lysate was added to 50μl of prewashed Protein G beads coated either with anti GRP78 antibody or rabbit IgG and incubated at 4°C for 4h with end to end mixing. Respective sperm lysates incubated with rabbit IgG coated Protein G beads served as negative control for IP experiment. The bound antigen- antibody complex was pelleted by centrifugation, the pellets washed thrice for 5min in NP40 lysis buffer and the proteins eluted in Laemmli buffer by heating at 100°C for 10minwere subjected to SDS PAGE followed by Western blot analysis using anti GRP78 antibody and Pan phospho antibody (Invitrogen, Frederick, MD, US).

#### Immunoprecipitation of Phosphoproteins

Phosphoproteins from rat testicular- and caudal- sperm lysates were immunoprecipitated using agarose beads conjugated anti- Phosphoserine(Clone PSR-45), anti- Phosphotyrosine(Clone PT- 66)and anti- Phosphothreonine (Clone PTR- 8) mouse monoclonal antibodies or unconjugated agarose beads (Sigma Aldrich, St. Louis, MO, USA). Briefly, 50μl of unconjugated agarose beads or the respective antibody conjugated agarose beads were incubated with 200μg of the protein lysate at 4°C for 4h with end to end mixing in the tube rotator. Post incubation, the beads were pelleted by centrifugation at 12000g for 5 min at 4°C and washed thrice in NP40 lysis buffer. The phosphoproteins bound to the respective antibodies were eluted in Laemelli buffer by heating at 95°C for 5min. The eluates so obtained were electrophoresed on a 12% SDS PAGE; transblotted onto a NC membrane and Western blot analysis for GRP78 was performed as described above. Unconjugated agarose beads used to account for any non-specifically binding proteins served as ‘Beads only’ control for the IP.

### Nanofluidic Proteomic Immunoassay (NIA)

GRP78 phosphorylation in sperm was deciphered by NIA using the NanoPro^TM^ 100 12 capillary system which allows simultaneous analysis of 12 samples (Protein Simple, Santa Clara, CA). NIA is a capillary based iso-electric focusing immunoassay. In this assay the proteins are resolved on the basis of their isoelectric points (pI) in the capillaries, UV immobilized, and after immunoprobing the signals are detected by a chemiluminescent substrate. The chemiluminescent signal is represented as a chemiluminescence isoelectropherogram of relative luminescence units (RLU) on the Y axis versus isoelectric point (pI) on the X axis.

20ng of protein lysates from rat- or human- sperm were mixed with Sample diluent, Premix G2 pH 5–6 separation gradient and fluorescent pI standard ladder 4 (Protein Simple, Santa Clara, CA)and iso-electric focused. Post separation, the proteins were UV immobilized and probed with the polyclonal anti-GRP78 antibody (1:50) which was detected using HRP conjugated swine anti-rabbit IgG (1:100) and the signals visualized by ECL were captured by a CCD camera. The digital images were analyzed and quantified using Compass software (Cell Biosciences, Santa Clara, CA).

### Phosphatase Assays

Phosphatase assays were carried out using λ-PP and CIP (New England Biolabs, Ipswich, MA) to determine the phosphoforms of GRP78 in rat- and human- sperm. The conditions for phosphatase assays were standardized for caudal- and testicular- sperm proteins (S5 Fig). Accordingly, for λ-PP assay, 100μg of rat- or human- sperm protein lysates were incubated without or with 300U of enzyme at 30°C for 2h. For CIP assay, 100μg of protein lysates were incubated without or with 32U of enzyme at 37°C for 2h in case ofrat sperm lysate and for 2h and overnight in case of human sperm lysate. Post phosphatase treatment, these lysates were analyzed for GRP78 peaks by NIA.

### Statistical analyses

Statistical analyses were done using Paired Students’‘t’ test for phosphatase assays and Unpaired Students’ ‘t’ test for all other analyses. Values are expressed as mean ± SD. The significance level was set at P ≤ 0.05.

## Results

### Differential expression of phosphorylated GRP78 in asthenozoosperm by DIGE

By DIGE only the abundant proteins could be identified. Hence subsequently we used nano UPLC-MS^E^ for identifying the phosphoproteins differentially expressed in asthenozoosperm, data of which has already been published. GRP78 was one of the proteins observed to be significantly reduced by this approach [12]. By DIGE coupled with DeCyder analysis one of the abundant proteins that were identified was GRP78. Phosphorylated GRP78 was observed to be over six fold reduced in asthenozoosperm. A shift in position of the GRP78 spot towards the basic side was observed in asthenozoosperm compared to that in normal sperm (Fig 1B). This suggests that phosphorylation is less in asthenozoosperm compared to normal sperm.

### Expression of GRP78 in human and rat sperm

**Fig 2:**
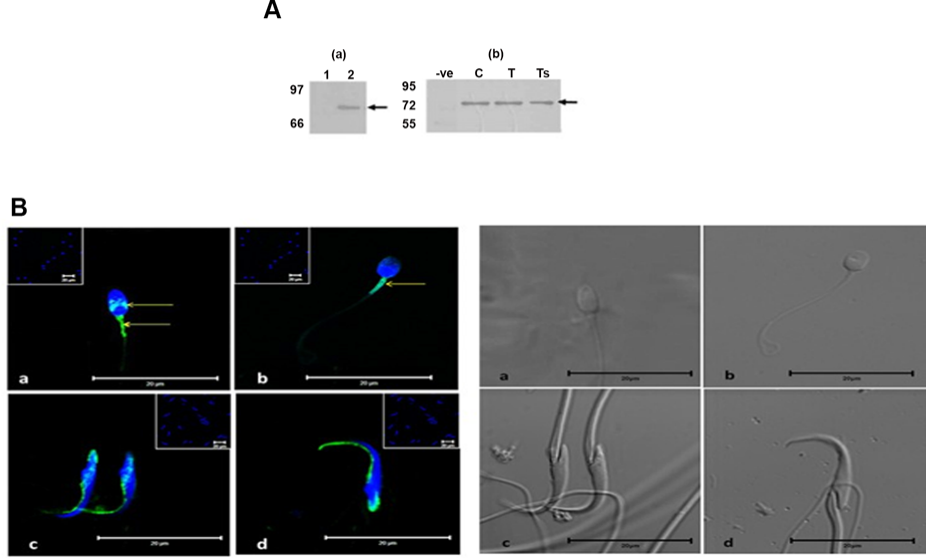
GRP78 expression in sperm. A) Western blot analysis of GRP78 in human sperm, rat testis, and rat testicular- and caudal- sperm. GRP78 expression in human sperm (a) [Lane 1: negative control], and rat sperm and testis (b) [-ve: Negative control, C: Caudal sperm, T: Testicular sperm, Ts: Testicular tissue] B) IIF localization of GRP78. In normal human sperm, GRP78 expression seen in the equatorial region of sperm head, neck and midpiece when non-permeabilized (a) and on neck and midpiece when permeabilized (b). In caudal (mature) rat sperm, GRP78 localization is observed in the equatorial region of sperm head when non-permeabilized (c) and on neck and anterior tip of sperm head on permeabilization (d) (Left panel). Inset shows the negative control for the respective images. DIC images of the respective sperm (Right panel)

Western blot analysis using GRP78 antibody demonstrated a band of ~78kDa in human sperm [Fig 2A (a)]. It was also detected in rat testicular tissue, and in testicular-, and caudal- sperm [Fig 2A (b)]. By Indirect Immunofluorescence using non permeabilized normal human sperm, GRP78 expression was seen in the equatorial region of sperm head, neck and mid- and on neck and mid-piece when permeabilized using 1% Triton X- 100 and 0.5% glacial acetic acid[Fig 2B (a, b)]. In rat caudal (mature) sperm, GRP78 localization is observed in the equatorial region of sperm head when non-permeabilized and on neck and anterior tip of sperm head on permeabilization [Fig 2B (c, d)].

### GRP78 is phosphorylated at serine, threonine and tyrosine residues in sperm

**Fig 3:**
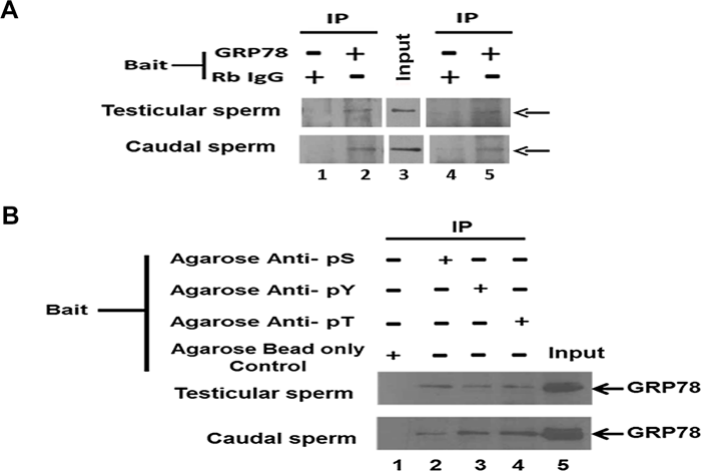
GRP78 phosphorylation in rat testicular- and caudal sperm. (A) GRP78 Immunoprecipitation. GRP78 immunoprecipitated from rat testicular- and caudal-sperm lysates was electrophoresed on 12% SDS-PAGE, transblotted and probed with Anti- GRP78 antibody(Lane 1–2) and Pan Phospho antibody (Lane 4–5). Lane 3: Input which was probed with Anti- GRP78 antibody. Rabbit IgG served as an isotype control for IP.**(B) Agarose bead Immunoprecipitation of Phosphoproteins.** Phosphoproteins were immunoprecipitated from 200μg of testicular-, or caudal sperm- lysates using either unconjugated agarose beads (control) or agarose beads conjugated anti Phospho-antibodies, electrophoresed on a 12% SDS-PAGE, transblotted and the blots were probed with Anti- GRP78 antibody. Lane 1: Control. Lanes 2–4: anti- Phosphoserine (anti- pS), anti- Phosphotyrosine (anti- pY), and anti- Phosphothreonine (anti- pT), respectively; Lane 5: Input.

Immunoprecipitated GRP78 probed with a polyclonal Pan phospho antibody reveals that GRP78 is phosphorylated in rat testicular- and caudal- sperm (Fig 3A). Band seen in lane 2 represents GRP78 and that seen in lane 5 indicates its phosphorylation. A very weak band is also seen in lane 4. Phosphoproteins from rat sperm lysates immunoprecipitated using anti -Phosphoserine, -Phosphotyrosine and -Phosphothreonine antibodies when probed with GRP78 antibody shows GRP78 to be phosphorylated at serine, threonine and tyrosine residues in both testicular- as well as in caudal- sperm (Fig 3B). Proteins immunoprecipitated using unconjugated agarose beads when probed with GRP78 antibody does not show the GRP78 band.

### Multiple forms of GRP78 exist in sperm

**Fig 4:**
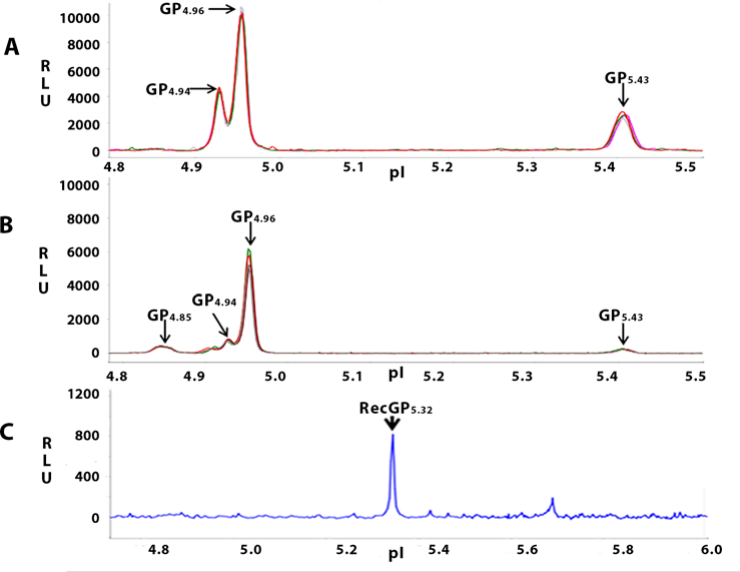
NIA profile of GRP78 in rat testicular- and cauda epididymal sperm. NIA for GRP78 was performed in 20ng of the respective sperm lysates in Premix of pI range 5–6 and spiked with pI standard 4 as the standard ladder. Figure shows a representative isoelectropherogram of GRP78 in rat testicular- and caudal- sperm (4 replicates represented by green, pink, red and grey peaks). Three peaks GP_4.94_, GP_4.96_ and GP_5.43_, are observed for testicular sperm (**A**). For caudal sperm, an additional peak GP_4.85_ is also observed (**B**). The pI of recombinant GRP78 is observed to be 5.32 (**C**) ‘GP’ represents the GRP78 peak, and the value in subscript indicates the pI of the respective peak. Experiments were performed thrice with 3 biological replicates and a minimum of 4 technical replicates for each.

The expanse of phosphorylation of GRP78 in rat- and human- sperm and its differential phosphorylation in asthenozoosperm was discerned by Nanofluidic Proteomic Immunoassay (NIA). Assays for GRP78 phospho-detection in sperm were first standardized, detailed description of which has been provided (S1 Text). The optimization data can be found in the supplemental information data (S1 to S4 Figs). Following the optimized conditions, GRP78 was analyzed from 20ngof testicular- or caudal- sperm lysates resolved in the pI range of 5–6. Three peaks of GRP78 at pI 4.94 (GP_4.94_), 4.96 (GP_4.96_) and 5.43 (GP_5.43_) were consistently observed for testicular sperm. For caudal sperm, four peaks at pI 4.85 (GP_4.85_), 4.94 (GP_4.94_), 4.96 (GP_4.96_) and 5.43 (GP_5.43_) were observed (Fig 4A and 4B). To identify the peak representing unphosphorylated GRP78, 0.5pg recombinant GRP78 (StressMarq Biosciences, Victoria, Canada) was similarly resolved by NIA. The pI of recombinant GRP78 was observed to be 5.32 (Fig 4C).

### Differential phosphorylation of GRP78 in testicular and caudal sperm

**Fig 5:**
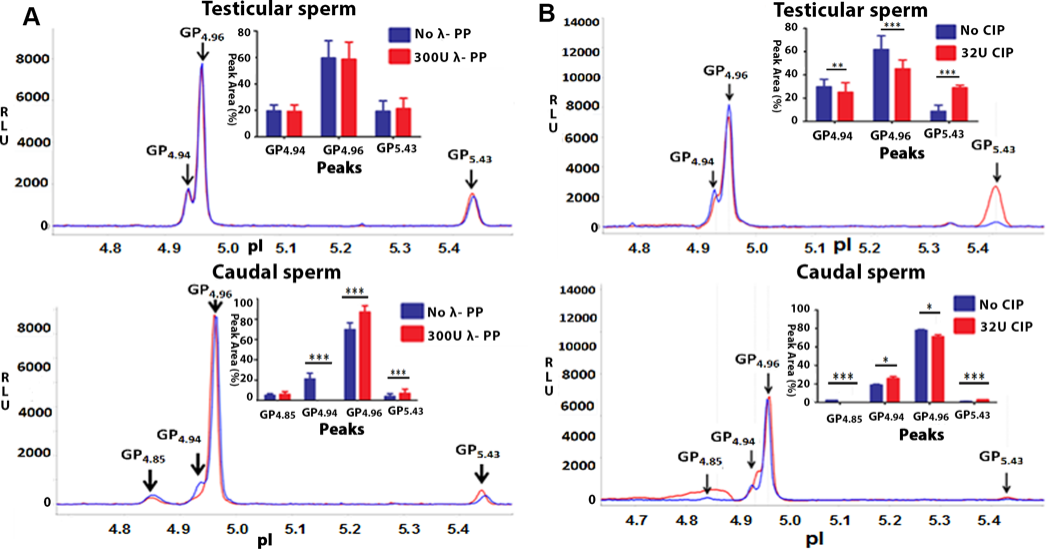
Phosphorylated forms of GRP78 in rat testicular- and caudal sperm. 100μg of testicular- or caudal- sperm protein was incubated for 2h without or with 300U of λ-PP at 30°C (**A**), or without or with 32U of CIP at 37°C (**B**) followed by NIA using 20ng of protein lysate thus treated to detect the GRP78 peaks. Figure shows representative isoelectropherograms for testicular- (Upper panel) and caudal- sperm (Lower panel). Figures in inset represent bar diagrams of the cumulative data. Values are mean ± Standard Deviation (SD). Statistical significance was determined using Paired Students’ ‘t’ test and significance level set at P ≤ 0.05.

To determine whether the different forms of GRP78 represented the different phosphorylation states of GRP78, Phosphatase assays were done. Incubation of testicular sperm with λ-PP demonstrated no change in any of the peaks (Fig 5A- Upper panel). The P values were0.19, 0.23 and 0.07 for GP_4.94_, GP_4.96_ and GP_5.43_, respectively. With caudal sperm, post λ-PP reaction, no change was observed in GP_4.85_ (P = 0.10), whereas GP_4.94_ showed complete reduction indicating this might be a phosphorylated form of GRP78 (P = 3.85 x 10^−11^). Significant increase was observed in GP_4.96_ (P = 3.5 x 10^−11^) and in GP_5.43_ (P = 5.4 x 10^−5^) (Fig 5A- Lower panel). Experiments were performed in 7–8 biological replicates with atleast 2 technical replicates for each. As complete dephosphorylation was not observed in caudal sperm and absolutely no dephosphorylation was observed in testicular sperm, we investigated whether the presence of phosphatase inhibitors used in the NP40 lysis buffer in any way influenced the GRP78 peak profiles of λ-PP treated testicular and caudal sperm lysates. The peak profile was similar regardless ofthe presence or absence of phosphatase inhibitors in the lysis buffer(S4 Fig). On CIP treatment of testicular sperm, peaks GP_4.94_ [P = 0.003] and GP_4.96_ [P = 0.0003] were significantly reduced indicating that these are indeed the phosphorylated forms of GRP78. GP_5.43_ [P = 0.0009] was significantly increased (Fig 5B-Upper panel). With caudal sperm, GP_4.85_ showed complete reduction [P = 7.02 x 10^−6^] and GP_4.96_ showed partial yet significant reduction [P = 0.02] indicating that these peak are phosphorylated forms of GRP78. Significant increase was observed in GP_4.94_ [P = 0.016] and in GP_5.43_ [P = 9.89 x 10^−7^], (Fig 5B–Lower panel). These assays were performed in two biological replicates with triplicates for each. Based on these observations, we believe that three phosphorylated forms of GRP78 are present in caudal sperm while in testicular sperm two phosphorylated forms exist. The observed pI for unphosphorylated GRP78 is 5.43.

### Peaks GP_4.94_, GP_4.96_ and GP_5.04_ represent phosphorylated forms of GRP78 in the human sperm

**Fig 6:**
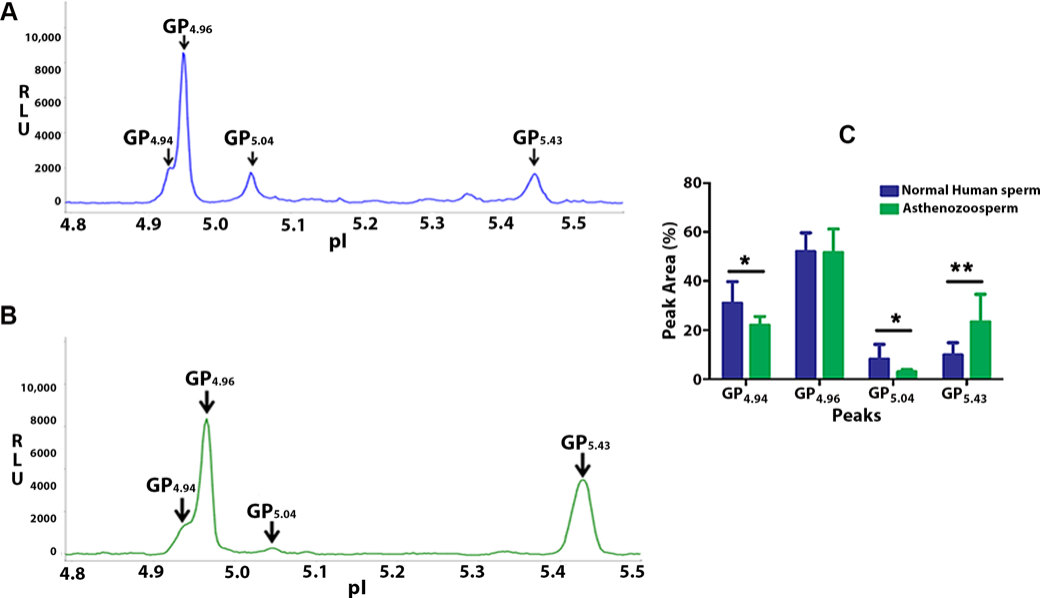
Multipleforms of GRP78 in Human sperm by NIA. Representative isoelectropherograms of NIA profile for GRP78 in normal sperm **(A)** and asthenozoosperm **(B)**. Four peaks GP_4.94_, GP_4.96_^,^ GP_5.04_ and GP_5.43_ are consistently observed. Bar diagram of the cumulative data comparing GRP78 profile of Asthenozoosperm with that of Normozoosperm is shown **(C)**. Experiments were performed in 3 biological replicates for each group with 3 technical replicates for each. Values expressed are mean ± Standard Deviation (SD). Statistical significance was determined using Unpaired Students’ ‘t’ test with significance level set at P ≤ 0.05. ‘GP’ represents the GRP78 Peak and the value in the subscript indicates the pI of the respective peak.

**Fig 7:**
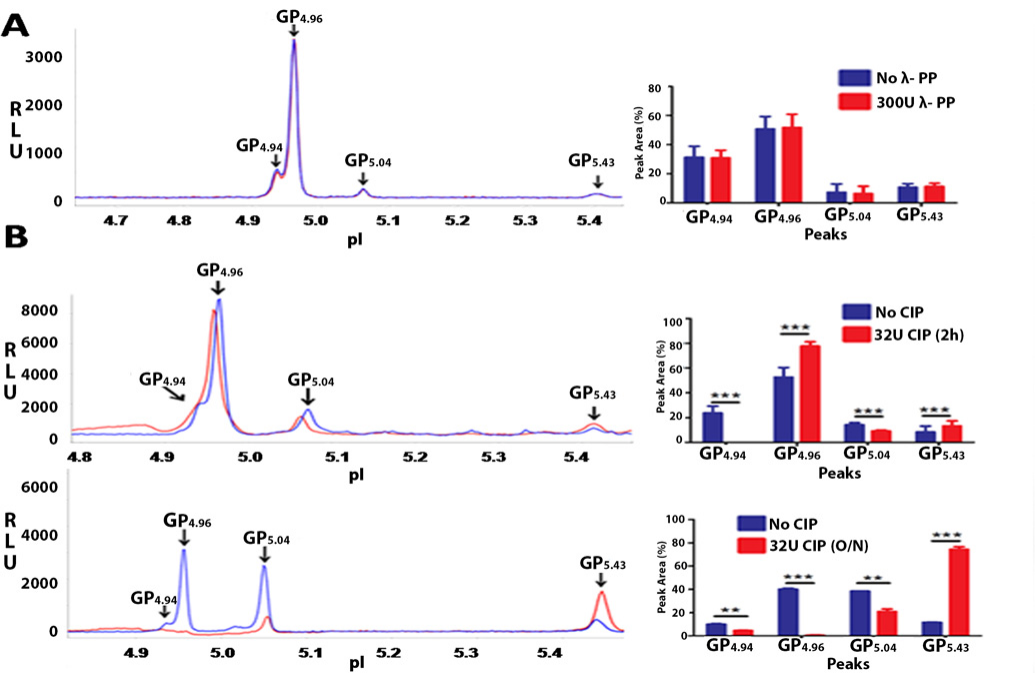
Phosphorylated forms of GRP78 in human sperm. Phosphorylated forms of GRP78 in human sperm were discerned using λ-PP and CIP. Figure shows representative isoelectropherograms of NIA profiles for Normal human sperm post λ-PP **(A)** and CIP **(B)**treatment. 100μg of human sperm protein was incubated without or with 300U of λ-PP for 2h at 30°C. For CIP treatment, reactions were incubated for 2h or overnight at 37°C. 20ng of this protein was used for NIA. No significant change was observed in the peak profile post λ-PP reaction **(A)**. On CIP treatment (2h incubation), complete reduction was observed in GP_4.94_ and significant reduction in GP_5.04_. Significant increase was observed in GP_4.96_ and GP_5.43_ **[Fig B; upper panel].**On overnight incubations with CIP, significant reduction was observed in GP_4.94_ and GP_5.04_. GP_4.96_ was completely reduced and a significant increase was observed in the unphosphorylated form GP_5.43_ **[Fig B; lower panel].** Values are expressed as mean ± SD. Graphical representations of the cumulative data for GRP78 peak profiles in sperm post λ-PP / CIP treatments are shown.

The GRP78 profile for human sperm consistently showed 4 peaks GP_4.94_, GP_4.96_, GP_5.04_ and GP_5.43_ (Fig 6A and 6B). Analysis of the GRP78 profile of asthenozoosperm and normal sperm revealed that peaks GP_4.94_ [P = 0.014] and GP_5.04_ [P = 0.02] are significantly reduced in asthenozoosperm. GP_5.43_ wassignificantly increased [P = 0.003] in asthenozoosperm compared to normal sperm. GP_4.96_ was comparable in asthenozoosperm and normal sperm [P = 0.93] (Fig 6C). λ-PP treatment of normal sperm shows no reduction in any of the peaks (Fig 7A)**.** CIP treatment revealed that GP_4.94_, GP_4.96_ and GP_5.04_ are the phosphorylated forms of GRP78 (Fig 7B). Incubations with CIP for 2h showed complete reduction in GP_4.94_ [P = 0.0001] and significant decrease in GP_5.04_ [P = 0.0001]. Significant increase was observed in GP_4.96_ [P = 0.00005] and in GP_5.43_[P = 0.0006] (Fig 7B; Upper panel). On overnight incubations, significant reduction was observed in GP_4.94_ [P = 0.007] and GP_5.04_ [P = 0.005]. GP_4.96_ [P = 0.0003] showed complete reduction and significant increase was observed in GP_5.43_ [P = 0.0004] (B; Lower panel). Experiments were performed in 2 biological replicates with 3 technical replicates for each. Based on these phosphatase assays, we infer that GP_4.94_, GP_4.96_and GP_5.04_ are the three phosphorylated forms of GRP78 in human sperm. Reduction of peak intensities on CIP treatment suggests that in human sperm GRP78 may be predominantly tyrosine phosphorylated.

## Discussion

Sperm motility is indispensable for normal procreation and poor sperm motility remains a principal cause of male infertility. Although it is being extensively researched, it remains an enigmatic phenomenon. Our efforts to understand this process, led us to the identification ofGRP78 as one of the few proteins differentially phosphorylated in asthenozoosperm. Phosphorylated GRP78 was over 2 fold decreased in asthenozoosperm compared to that in normozoosperm [12]. GRP78 (also known as HSPA5) and six members from four different HSP families are known to be putative residents of the human sperm surface [6]. GRP78 has been well characterized in macrophage motility[3, 5]. However the significance of its presence on sperm and its role in sperm function has not yet been deciphered.

We had earlier reported that phosphorylated GRP78 is significantly reduced in asthenozoosperm [12]. This has been reaffirmed by DIGE (Fig 1B).

GRP78 expression has been reported in the neck region and on the surface of human sperm [6, 8]. We observed GRP78 localization in the equatorial region of the sperm head in rat and human nonpermeabilized sperm suggesting its surface expression (Fig 2B). Localisation in the neck and midpiece imply a role for GRP78 in calcium mobilization, as calcium stores have been reportedly present in the neck and mid-piece region [19].

In bovine and hamster liver, four isoforms of GRP78 have been observed and through Phosphoamino acid analysis, it is reportedly threonine phosphorylated [20]. GRP78 has been reported to be phosphorylated at serine and threonine residues in hamster fibroblast cells [21]. In human spermGRP78 is reportedly not tyrosine phosphorylated [8]. There is no information about the phosphorylation at serine or threonine residues. Our data shows that the protein is phosphorylated at serine, threonine and tyrosine residues in testicular-, as well as caudal- sperm of rat (Fig 3). A weak band was also observed for immunoprecipitates of rabbit IgG probed with Pan phospho antibody(Fig 3A, Lane 4). This weak band may represent another phosphoprotein immunoprecipitated by Rabbit IgG and recognised by the Pan phospho antibody. This band is certainly not that of GRP78 as the same immunoprecipitates did not show GRP78 band when probed with Anti GRP78 antibody (Lane 1).We further deciphered the phosphoforms of GRP78 by NIA. Three GRP78 specific peaks GP_4.94_, GP_4.96_ and GP_5.43_ were observed for testicular sperm. For caudal sperm, four peaks GP_4.85,_ GP_4.94_, GP_4.96_ and GP_5.43_ were observed (Fig 4A and 4B). For human sperm, four GRP78 peaks namely, GP_4.94_, GP_4.96_, GP_5.04_ and GP_5.43_ were observed (Fig 6A and 6B). Recombinant GRP78 (untagged) resolved at pI 5.32, indicating 5.32 as the pI of unphosphorylated GRP78 (Fig 4C). This is higher than the calculated pI of unphosphorylated GRP78 which is 5.07 as calculated by Phosphosite prediction tool [22]. This can be attributed to the semi native conditions used for NIA. We presumed the peak GP_5.43_to be that of unphosphorylated GRP78 as it was consistently observed for rat testicular- and caudal sperm as well as human sperm and very close to the observed pI of recombinant GRP78. This was confirmed by Phosphatase assays.

λ-PP is a serine/threonine- specific phosphatase [23], whereas, CIP dephosphorylates tyrosine residues [24]. The phosphorylation status of the identified peaks was discerned using these phosphatases (Figs 5 and 7).Post phosphatase assay using λ-PP in rat caudal sperm, GP_4.85_remained unchanged; 100% reduction of GP_4.94_ was observed and significant increase was observed in GP_4.96_ and GP_5.43_ (Fig 5A-Lower panel). The partial dephosphorylation of GP_4.94_ may account for the increase in peak area of GP_4.96_ whilst its complete dephosphorylation reflects as a significant increase in the peak area of unphosphorylated form of GRP78 (GP_5.43_). Interestingly, testicular sperm GRP78 forms GP_4.94_ and GP_4.96_ which were not altered on λ-PP treatment showed significant reduction with CIP treatment indicating that these are the phosphorylated forms of GRP78 and that they may be predominantly tyrosine phosphorylated. Intriguingly, our IP studies (Fig 3B) suggest that in testicular sperm, GRP78 is phosphorylated at all 3 (Ser, Thr and Tyr) residues. But this does not reflect in the NIA for testicular sperm treated with λ- PP (Fig 5A). There is an obvious discrepancy. This may likely be due to the nature and sensitivity of the two techniques namely IP and NIA as both these observations are consistent. On the basis of a) Agarose beads IP experiment, and b) Failure to obtain 100% reduction in peaks post incubation of testicular sperm protein with (32U) CIP enzyme, we believe that testicular sperm GRP78 is phosphorylated at Ser and Thr residues as well. Dephosphorylation of GP_4.94_ and GP_4.96_ reflected as an increase in the unphosphorylated form GP_5.43_ (Fig 5A and 5B-Upper panel). With caudal sperm, complete reduction of GP_4.85_and partial yet significant reduction of GP_4.96_ with CIP confirms that these peaks are phosphorylated forms of GRP78. Significant increase was observed in GP_4.94_ and in GP_5.43_. On the basis of responses to λ-PP and CIP, we believe that partial dephosphorylation of GP_4.85_ may reflect as an increase in the phosphorylated form GP_4.94_whereas its complete dephosphorylation and dephosphorylation of a few molecules of GP_4.96_ may have contributed to the increase in unphosphorylated form GP_5.43_ (Fig 5B-Lower panel). This prompts us to believe that in rat caudal sperm, the phosphorylated form GP_4.85_ and GP_4.96_ may be having more number of tyrosine residues phosphorylated whereas GP_4.94_, must have atleast one serine / threonine residue phosphorylated.

In mature human sperm GP_4.94_, GP_4.96_ and GP_5.04_ were not altered on λ-PP treatment (Fig 7A). However on treatment with CIP, a significant reduction was observed in GP_4.94_andGP_5.04_with increase inGP_4.96_andunphosphorylated GP_5.43_on 2h incubation. The increase inGP_4.96_can be attributed to partial dephosphorylation of GP_4.94_. When incubated O/N with CIP, GP_4.94,_ GP_4.96_ and GP_5.04_ show significant reduction with a concomitant increase in unphosphorylated GP_5.43_ (Fig 7B). These observations suggest that in mature human sperm, GP_4.94_, GP_4.96_ and GP_5.04_ are the phosphorylated forms of GRP78 and that GRP78 may be predominantly tyrosine phosphorylated.

Calculations using the Phosphosite prediction tool indicate that for GRP78 to have a pI of 5.04, 4.95, 4.93, and 4.86 it has to have 1, 4, 5 and 8 amino acids phosphorylated, respectively. The calculated pI for unphosphorylated GRP78 is 5.07. Our observations of GRP78 peaks at pI 5.04, 4.96, 4.94 and 4.85 suggest that these are the phosphorylated forms of GRP78 present in rat testicular-, and caudal sperm and ejaculated human sperm. GP_5.43_ represents unphosphorylated GRP78. This data also suggests that in mature rat sperm, possibly 4-8aa’s are phosphorylated in a GRP78 molecule whereas in mature human sperm GRP78 phosphorylation sites likely range from 1 to 5.

During their sojourn from the testis to the epididymal cauda, sperm undergo several changes which render them motile and functionally competent. Post translational modification such as phosphorylation plays a very important role in sperm maturation. The exclusivity of GP_4.85_ to the caudal sperm, suggests that GRP78 phosphorylation in sperm increases during its maturation. This is also evident from the significant decrease in the percentage of unphosphorylated GRP78 in caudal sperm vis-a-vis the testicular sperm. Increase in GRP78 phosphorylation during sperm maturation indicates GRP78 may play a pivotal role in sperm function. By Panphospho immunoprecipitation, we had observed GRP78 to be less phosphorylated in asthenozoospermic individuals as compared to normal and this has also been validated by DIGE (12) (Fig 1B). Our observation of significant reduction of phosphoforms GP_4.94_ and GP_5.04_ in asthenozoosperm provides a possible explanation for this and suggests that these forms may have a significant role in sperm function.

**Fig 8:**
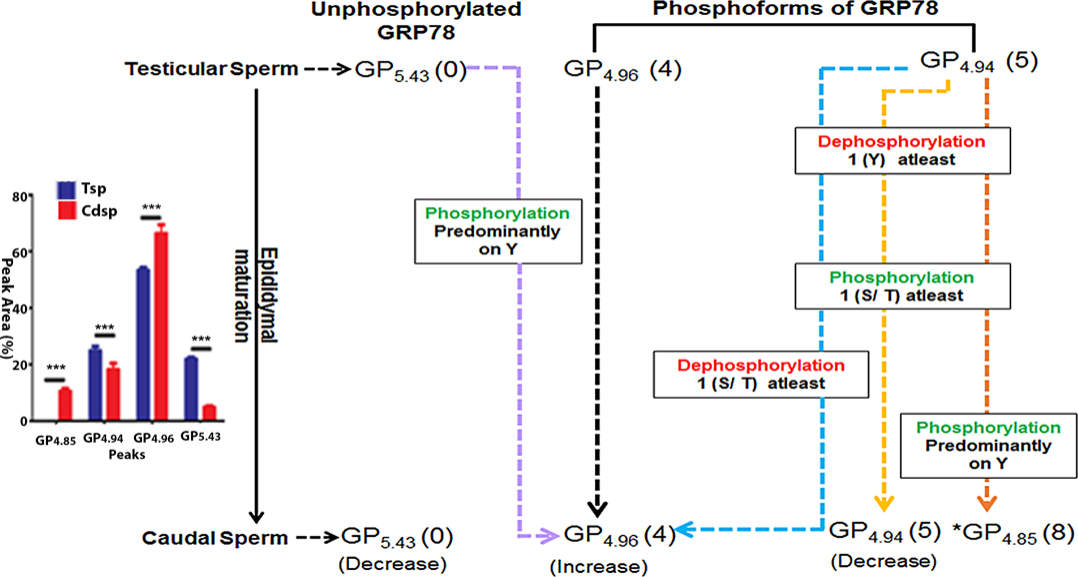
Phosphorylation changes in GRP78 during sperm maturation Immature testicular sperm are known to undergo structural and functional maturation in the epididymis. 2 phosphorylated forms of GRP78 were observed in testicular (immature) sperm (GP_4.94_ and GP_4.96_) and 3 phosphorylated forms in caudal (mature) sperm (*GP_4.85,_ GP_4.94_ and GP_4.96_). ‘*’ represents phosphorylated form of GRP78 exclusive to rat caudal sperm. Based on NIA profile and responses of rat testicular- and caudal—sperm GRP78 to phosphatases λ- PP and CIP, we hypothesize putative phosphorylation/ dephosphorylation changes in sperm GRP78 as the sperm traverses from the testis to caudal region of the epididymis and attains maturity. Testicular sperm represents immature sperm while the caudal sperm represents mature sperm. The remarkable changes are as follows;
GP_4.96_ responds similarly to phosphatase treatment in both immature and mature sperm suggesting that GP_4.96_ is retained in the same form in the mature sperm as present in immature sperm (Black dashed arrows).GP_4.96_ increases in the mature caudal sperm. This may be due to a) phosphorylation of GP_5.43_ (Purple dashed arrow) which is substantiated by the observed decrease in GP_5.43_ in the mature sperm, or b) phosphorylation changes on GP_4.94,_ in the immature sperm as it traverses through the epididymis (Blue dashed arrow).GP_4.94_ in mature sperm is probably not the same as in immature sperm as this form shows difference in response to phosphatase treatment in mature versus immature sperm indicating possible phosphorylation /dephosphorylation events happening in GP_4.94_ in immature sperm to give rise to new form of GP_4.94_ in mature sperm (Yellow dashed arrow).In the mature sperm, a) GP_4.85_ and GP_4.96_ are resistant to λ- PP but responsive to CIP, b) GP_4.94_ decreases significantly and a new GRP78 phosphoform GP_4.85_ emerges. Given that λ- PP is a serine / threonine phosphatase and CIP is a tyrosine specific phosphatase, we believe that GP_4.94_ in the immature sperm might undergo dephosphorylation on atleast 1 phosphorylated tyrosine(Y) residue, phosphorylation on atleast 1 serine/ threonine (S/ T) residue, and phosphorylation of predominantly tyrosine (Y) residues thus resulting in GP_4.85_ form in mature sperm (Orange dashed arrow).
Graph on the left shows comparison of the respective peaks in testicular- (Tsp) and caudal- sperm (Cdsp). Number in parentheses indicates the number of amino acids phosphorylated.

On the basis of substrate specificities of the enzymes used, we can infer that in caudal sperm, GP_4.94_ may be serine / threonine phosphorylated while in testicular sperm it may not have a serine or threonine residue phosphorylated as treatment with λ-PP demonstrated complete reduction of GP_4.94_ for caudal sperm but not for testicular sperm suggesting that the phosphoform GP_4.94_ may not be the same in testicular- and caudal- sperm. However this same peak showed a significant reduction post CIP treatment. Phosphorylation changes in GRP78 during sperm maturation as decoded by us from our data are presented as a schematic illustration (Fig 8).

To summarize, our study provides novel information on the spatial changes inGRP78 phosphorylation during sperm maturation. By NIA, we have deciphered GRP78 phosphoforms in rat- and human- sperm that have been hitherto unreported. Our observations reveal that in rat, GP_4.96_, GP_4.94_ and GP_4.85_ are the three phosphorylated forms of GRP78 seen in the mature sperm as against two phosphorylated forms GP_4.96_ and GP_4.94_in immature sperm, indicating increase in GRP78 phosphorylation in sperm during epididymal maturation. In mature human sperm GP_5.04_, GP_4.96_, and GP_4.94_are the 3 phosphoforms observed; of these three phosphoforms, GP_4.94_ and GP_5.04_ are significantly reduced in asthenozoosperm suggesting a putative role of GRP78 phosphorylation in sperm function. The precise role that these may play in sperm motility can only be deciphered once the phosphorylation sites on GRP78 in sperm are identified. Presently studies are ongoing to decode the functional relevance of these forms.

## Supporting Information

S1 Text Optimization assays for GRP78 phosphodetection in sperm. (DOCX)

S1 Fig NIA profile of GRP78 in rat sperm using Premix of pI range 5–6. A) A representative isoelectropherogram of GRP78 profile in testicular sperm (red). Three peaks GP_4.94_, GP_4.96_ and GP_5.43_ were consistently observed for testicular sperm. In caudal epididymal sperm (blue), four peaks GP_4.85,_ GP_4.94_, GP_4.96_ and GP_5.43_ were consistently observed. ‘GP’ represents the GRP78 Peak and the value in the subscript indicates the pI of the respective peak. A negative control with no primary antibody did not show any peaks (green). B) Sensitivity of GRP78 profile detection by NIA. Caudal sperm lysates ranging from 40–2.5ng were used to determine the sensitivity of GRP78 detection. Figure depicts overlaid image of GRP78 profiles for caudal sperm at different concentrations of protein lysate. The colour codes for the different concentrations used, is shown in the table (Inset). A maximum of 4 peaks were observed at 20ng (red) and 40ng (green) of the lysate. Minimum 3 peaks were observed at all lower concentrations of the lysate. Hence lysate concentration of 20ng was used for the subsequent experiments. Inset shows the same observations in a tabular form. Experiments were performed using 2 biological replicates with 2 technical replicates for each.
(TIF)

S2 Fig Effect of Urea on the GRP78 profile in rat testicular- and caudal sperm. A representative isoelectropherogram of overlaid profiles of GRP78 for testicular sperm (A) and Caudal sperm (B) using 20ng of protein lysate made in lysis buffer containing No Urea (blue), 6M (red), and 9M (green) of Urea. Three peaks were observed for testicular- and four peaks for caudal sperm consistently, at all concentrations of Urea. The peak pI were the same as obtained using the respective lysates prepared in NP40 lysis buffer without Urea. This indicates that the peaks so obtained were specific to GRP78 and not a result of the cumulative pI which might be obtained because of the GRP78 interacting with its partners. With increase in Urea concentration, a decrease in peak intensity can be seen. Experiments were performed using 2 biological replicates with 2 technical replicates for each.
(TIF)

S3 Fig Effect of different concentrations of λ-PP on GRP78 peak profile in rat testicular- and cauda epididymal sperm. Representative isoelectropherogram depicting overlaid images of NIA profiles for testicular- (A) and caudal sperm (B) treated without or with 100–500 U λ-PP. 100 μg of testicular- or caudal sperm protein was incubated without (red) or with 100U (blue), 300U (green), or 500U (grey) of λ-PP for 2h at 30°C. Lysates with no λ-PP acted as control for the reaction. 20ng of sperm protein lysate was used in the NIA. No change is observed in any of the peak post phosphatase reaction in testicular sperm (A). For caudal sperm, complete reduction of GP_4.94_ was observed on incubation with 300U (P = 0.01) and 500U (P = 0.01) enzyme; a partial but significant reduction of this peak was seen on incubation with 100U (P = 0.03) of the enzyme. Peak percent area for GP_4.96_ was significantly higher post phosphatase reaction at all concentrations of the enzyme whereas GP_5.43_ remained unchanged (B). Graphical representation of the data is shown in the inset. (C) Temporal effect of λ-PP on the peak profile was determined by incubating 100 μg of caudal sperm protein without (blue) or with 300U of λ-PP (red) at 30°C for 2 or 4h. Representative figures depicting overlaid images of NIA profiles for caudal sperm post λ-PP treatment for 2h (C) and 4h (D) are shown. On treatment with λ-PP for 2h, no change was observed in GP_4.85_ (P = 0.10), whereas GP_4.94_ was significantly reduced (P = 0.003). A significant increase was observed in GP_4.96_ (P = 0.003) and GP_5.43_ (P = 0.02) (C). Post 4h of λ-PP treatment, significant increase was observed in GP_4.85,_ (P = 0.014). GP_4.96_ (P = 0.003) and GP_5.43_ (P = 0.011) whereas GP_4.94_ showed significant reduction (P = 0.003) (D). Insets show graphical representations of the same. All values are expressed as mean ± SD. Experiments were performed using 2 biological replicates with 2 technical replicates for each.
(TIF)

S4 Fig Effect of Phosphatase Inhibitors present in NP40 lysis buffer on the outcome of λ-PP treatment of rat sperm lysates. This was studied by using testicular- or caudal sperm lysates prepared in NP40 lysis buffer containing phosphatase inhibitor cocktail or devoid of it, and incubating 100 μg of these lysates without or with 300U of λ-PP at 30°C for 2 h. NIA profiles obtained for GRP78 post λ-PP assay using testicular sperm lysate prepared in NP40 lysis buffer without the phosphatase inhibitor cocktail (A) yielded results similar to that seen in presence of phosphatase inhibitor cocktail in lysis buffer (B). These results indicate that phosphatase inhibitors in the lysis buffer at the concentration used does not influence λ-PP activity. GRP78 profiles post λ-PP assay for caudal sperm lysates prepared in NP40 lysis buffer without (C) or with (D) the phosphatase inhibitor cocktail, showed the same results. Graphical representations of the cumulative data are shown in inset. Values are expressed as mean ± SD. Experiments were performed using 2 biological replicates with 2 technical replicates for each.
(TIF)

## Abbreviations

BCA: Bicinchoninic acid
CIP: Calf intestinal alkaline phosphatase
HRP: Horseradish peroxidase
IIF: Indirect Immunofluorescence
Met and Cys: Methionine and Cysteine
Nano UPLC- MS^E^: Nano Ultra performance liquid chromatography–mass spectrometry Elevated Energy
NIA: Nanofluidic Proteomic Immunoassay
NP40: Nonylphenoxypolyethoxylethanol
PBS: Phosphate buffered saline
PBS-T: PBS containing Tween 20
PFA: Paraformaldehyde
SDS- PAGE: Sodium Dodecyl sulphate- Polyacrylamide gel electrophoresis
TMB- H_2_O_2_: Hydrogen Peroxide 3, 3’, 5, 5’ Tetramethylbenzidine
λ- PP: Lambda Protein Phosphatase
