# Supplementary material for: Glucose Regulated Protein 78 Phosphorylation in Sperm Undergoes Dynamic Changes during Maturation
Source: PLoS One. 2015 Nov 30;10(11):e0141858. doi: 10.1371/journal.pone.0141858 (PMC4664250; doi:10.1371/journal.pone.0141858)
Supplement: S1 Text — (DOCX) [file pone.0141858.s001.docx]

**Supporting Information**

**S1 Text. Optimization assays for GRP78 phosphodetection in sperm.** In order to optimize Nanofluidic Proteomic Immunoassay (NIA) for GRP78 in rat sperm, a basic screening protocol was carried out using a wide range separation gradient of pI (3-10). Since most of the peaks were observed in the pI range of 5-6, a separation gradient of pI (5-6) was used for subsequent experiments. Resolution of GRP78 peaks for testicular- and caudal sperm in the pI gradient 5-6 is shown in S1AFig. The negative control devoid of the primary antibody shows no peaks. The optimal protein concentration required for detecting GRP78 specific peaks was determined by performing NIA with 5 different concentrations ranging from 40 - 2.5 ng of caudal sperm protein lysate. Maximum numbers of peaks were consistently observed with 20 and 40ng of protein (S1B Fig). Hence for subsequent experiments, separation gradient of pI 5-6 with 20ng of protein was used. Since NIA is a semi native assay, there is a possibility that GRP78 may remain bound to its interacting partners and therefore the peak profile obtained may represent a cumulative pI of GRP78 and its interacting partners and not reflect forms of GRP78 alone. To test this possibility, urea (6M and 9M) was added in the sample lysis buffer to break the bonds, if any, between GRP78 and its interacting partners and NIA was carried out using both rat testicular- and caudal sperm lysates thus prepared. On addition of urea, no shift in pI was observed and only the peak intensity decreased, indicating the peaks obtained in the NIA profile of rat testicular and caudal sperm are indeed of GRP78 and its phosphorylated forms (S2A and B Fig). The reduced peak size with increasing concentrations of urea may be due to the changes in conformation induced by urea which consequently may have affected the GRP78 antibody binding to it. Having identified that the peaks obtained indeed reflected different forms of GRP78, we had to interpret what these peaks meant. To determine whether the peaks reflected phosphorylated forms of GRP78, serine/threonine-specific and tyrosine-specific phosphatases, λ-PP and CIP were used. The concentration of λ-PP for the phosphatase reaction was standardized using 100-500U of λ-PP on rat testicular- and caudal sperm lysate. No change was observed in the GRP78 profile for testicular sperm even at 500U of λ-PP enzyme (S3A Fig). With caudal sperm, dephosphorylating effect of λ-PP were visible and significant at all the three concentrations, albeit it was slightly less at 100U of the enzyme; 300U and 500U of λ-PP were equally effective (S3B Fig). The period of incubation with λ-PP was optimized using 300U of the enzyme. The enzyme was equally effective at 2, and 4h of incubation (S3C, D Fig). Hence for subsequent phosphatase experiments with λ-PP, 300U of the enzyme was used and incubations were done for 2h. Post Phosphatase assays using λ-PP, no change was observed in rat testicular sperm GRP78 profile and 100% dephosphorylation was not observed in caudal sperm. We therefore wanted to discern whether this was due to the phosphatase inhibitors present in the sample lysis buffer. Hence lysates were prepared in lysis buffer without phosphatase inhibitors. However there was no change in the GRP78 profiles of testicular- or caudal sperm lysates prepared in the lysis buffer containing phosphatase inhibitors and devoid of it (S4 Fig).
