# Supplementary figures and images for: Glucose Regulated Protein 78 Phosphorylation in Sperm Undergoes Dynamic Changes during Maturation
Source: PLoS One. 2015 Nov 30;10(11):e0141858. doi: 10.1371/journal.pone.0141858 (PMC4664250; doi:10.1371/journal.pone.0141858)

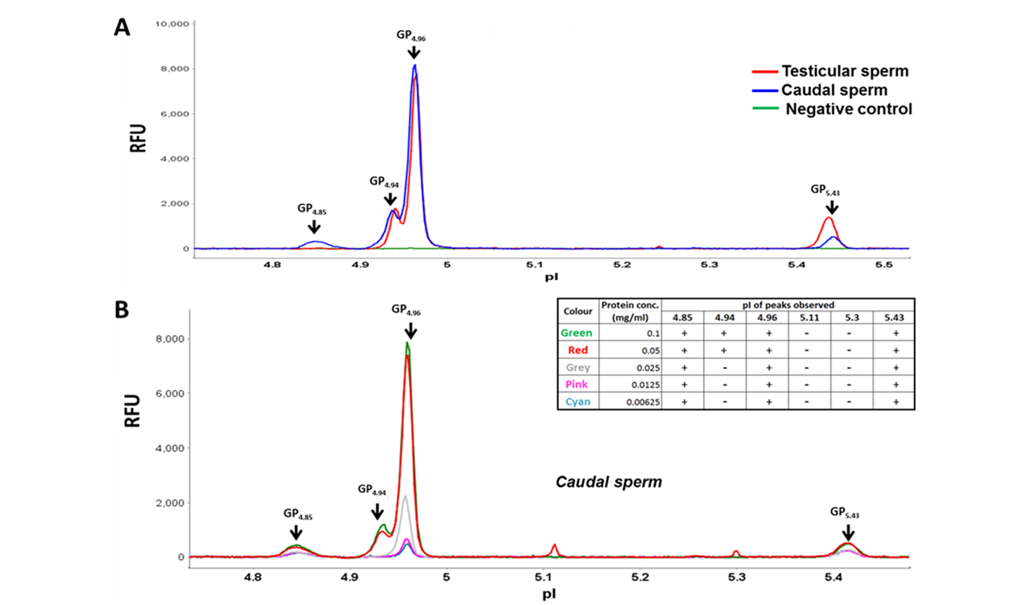

Supplement: S1 Fig — A) A representative isoelectropherogram of GRP78 profile in testicular sperm (red). Three peaks GP4.94, GP4.96 and GP5.43 were consistently observed for testicular sperm. In caudal epididymal sperm (blue), four peaks GP4.85, GP4.94, GP4.96 and GP5.43 were consistently observed. ‘GP’ represents the GRP78 Peak and the value in the subscript indicates the pI of the respective peak. A negative control with no primary antibody did not show any peaks (green). B) Sensitivity of GRP78 profile detection by NIA. Caudal sperm lysates ranging from 40–2.5ng were used to determine the sensitivity of GRP78 detection. Figure depicts overlaid image of GRP78 profiles for caudal sperm at different concentrations of protein lysate. The colour codes for the different concentrations used, is shown in the table (Inset). A maximum of 4 peaks were observed at 20ng (red) and 40ng (green) of the lysate. Minimum 3 peaks were observed at all lower concentrations of the lysate. Hence lysate concentration of 20ng was used for the subsequent experiments. Inset shows the same observations in a tabular form. Experiments were performed using 2 biological replicates with 2 technical replicates for each. (TIF) [file pone.0141858.s002.tif]

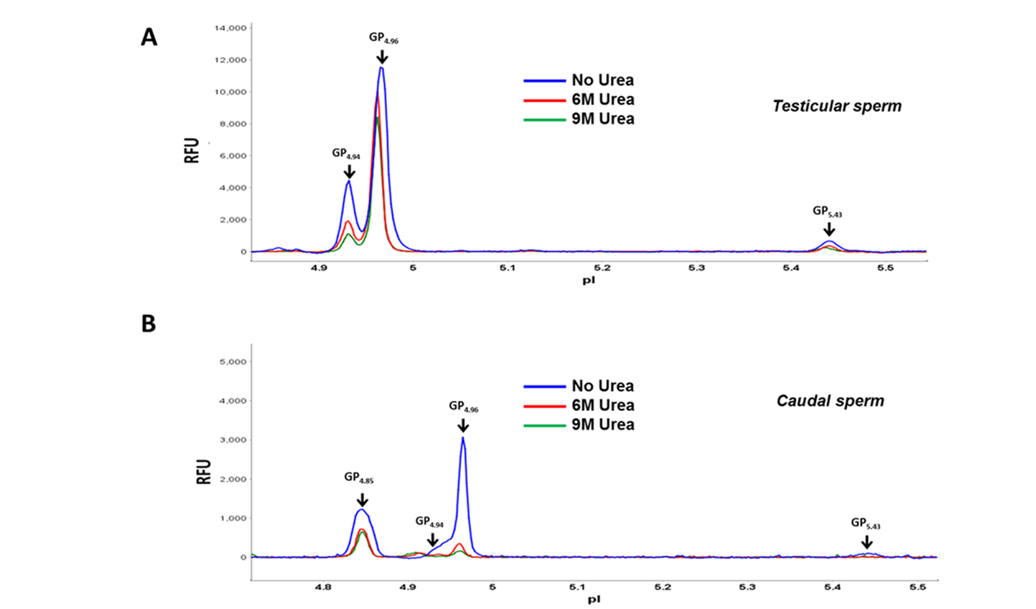

Supplement: S2 Fig — A representative isoelectropherogram of overlaid profiles of GRP78 for testicular sperm (A) and Caudal sperm (B) using 20ng of protein lysate made in lysis buffer containing No Urea (blue), 6M (red), and 9M (green) of Urea. Three peaks were observed for testicular- and four peaks for caudal sperm consistently, at all concentrations of Urea. The peak pI were the same as obtained using the respective lysates prepared in NP40 lysis buffer without Urea. This indicates that the peaks so obtained were specific to GRP78 and not a result of the cumulative pI which might be obtained because of the GRP78 interacting with its partners. With increase in Urea concentration, a decrease in peak intensity can be seen. Experiments were performed using 2 biological replicates with 2 technical replicates for each. (TIF) [file pone.0141858.s003.tif]

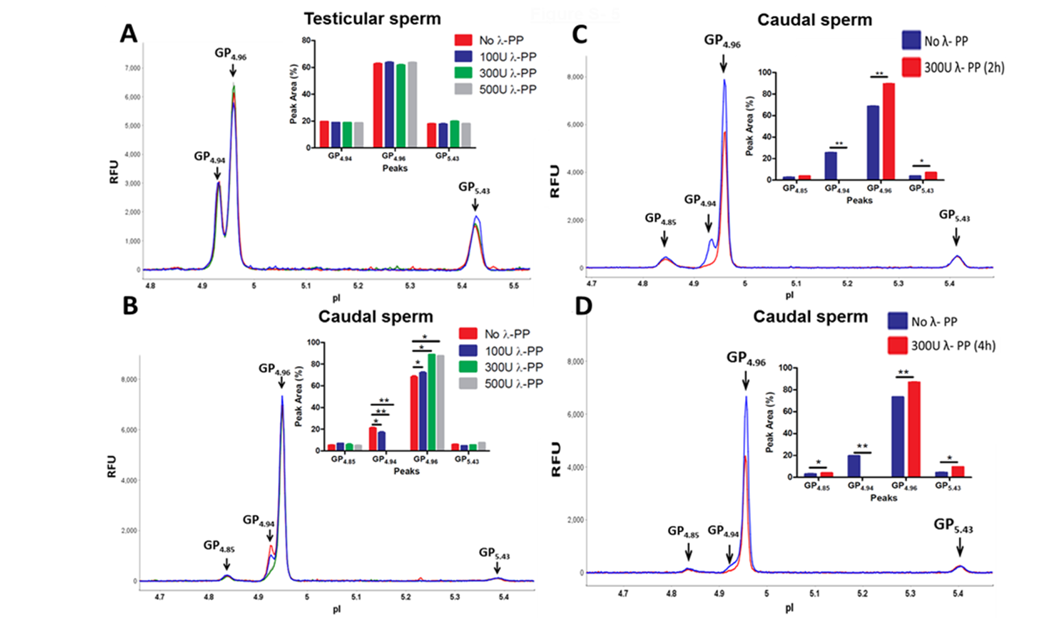

Supplement: S3 Fig — Representative isoelectropherogram depicting overlaid images of NIA profiles for testicular- (A) and caudal sperm (B) treated without or with 100–500 U λ-PP. 100 μg of testicular- or caudal sperm protein was incubated without (red) or with 100U (blue), 300U (green), or 500U (grey) of λ-PP for 2h at 30°C. Lysates with no λ-PP acted as control for the reaction. 20ng of sperm protein lysate was used in the NIA. No change is observed in any of the peak post phosphatase reaction in testicular sperm (A). For caudal sperm, complete reduction of GP4.94 was observed on incubation with 300U (P = 0.01) and 500U (P = 0.01) enzyme; a partial but significant reduction of this peak was seen on incubation with 100U (P = 0.03) of the enzyme. Peak percent area for GP4.96 was significantly higher post phosphatase reaction at all concentrations of the enzyme whereas GP5.43 remained unchanged (B). Graphical representation of the data is shown in the inset. (C) Temporal effect of λ-PP on the peak profile was determined by incubating 100 μg of caudal sperm protein without (blue) or with 300U of λ-PP (red) at 30°C for 2 or 4h. Representative figures depicting overlaid images of NIA profiles for caudal sperm post λ-PP treatment for 2h (C) and 4h (D) are shown. On treatment with λ-PP for 2h, no change was observed in GP4.85 (P = 0.10), whereas GP4.94 was significantly reduced (P = 0.003). A significant increase was observed in GP4.96 (P = 0.003) and GP5.43 (P = 0.02) (C). Post 4h of λ-PP treatment, significant increase was observed in GP4.85, (P = 0.014). GP4.96 (P = 0.003) and GP5.43 (P = 0.011) whereas GP4.94 showed significant reduction (P = 0.003) (D). Insets show graphical representations of the same. All values are expressed as mean ± SD. Experiments were performed using 2 biological replicates with 2 technical replicates for each. (TIF) [file pone.0141858.s004.tif]

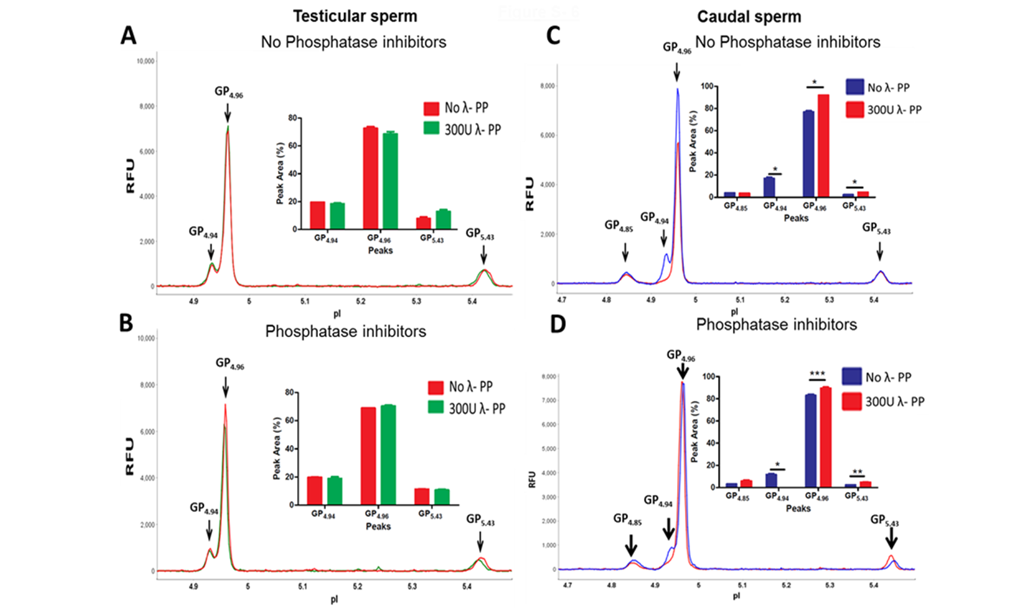

Supplement: S4 Fig — This was studied by using testicular- or caudal sperm lysates prepared in NP40 lysis buffer containing phosphatase inhibitor cocktail or devoid of it, and incubating 100 μg of these lysates without or with 300U of λ-PP at 30°C for 2 h. NIA profiles obtained for GRP78 post λ-PP assay using testicular sperm lysate prepared in NP40 lysis buffer without the phosphatase inhibitor cocktail (A) yielded results similar to that seen in presence of phosphatase inhibitor cocktail in lysis buffer (B). These results indicate that phosphatase inhibitors in the lysis buffer at the concentration used does not influence λ-PP activity. GRP78 profiles post λ-PP assay for caudal sperm lysates prepared in NP40 lysis buffer without (C) or with (D) the phosphatase inhibitor cocktail, showed the same results. Graphical representations of the cumulative data are shown in inset. Values are expressed as mean ± SD. Experiments were performed using 2 biological replicates with 2 technical replicates for each. (TIF) [file pone.0141858.s005.tif]
